# SARS-CoV-2 spike engagement of ACE2 primes S2′ site cleavage and fusion initiation

**DOI:** 10.1073/pnas.2111199119

**Published:** 2021-12-20

**Authors:** Shi Yu, Xu Zheng, Bingjie Zhou, Juan Li, Mengdan Chen, Rong Deng, Gary Wong, Dimitri Lavillette, Guangxun Meng

**Affiliations:** ^a^The Center for Microbes, Development and Health, Institut Pasteur of Shanghai, Chinese Academy of Sciences, University of Chinese Academy of Sciences, Shanghai 200031, China;; ^b^CAS Key Laboratory of Molecular Virology & Immunology, Institut Pasteur of Shanghai, Chinese Academy of Sciences, University of Chinese Academy of Sciences, Shanghai 200031, China;; ^c^Pasteurien College, Soochow University, Suzhou, Jiangsu 215006, China;; ^d^Nanjing Unicorn Academy of Innovation, Institut Pasteur of Shanghai, Chinese Academy of Sciences, Nanjing, Jiangsu 211135, China

**Keywords:** SARS-CoV-2 spike, spike protein, ACE2, membrane fusion, S2′

## Abstract

The COVID-19 pandemic caused by severe acute respiratory syndrome coronavirus 2 (SARS-CoV-2) infection has resulted in tremendous loss worldwide. Although viral spike (S) protein binding of angiotensin-converting enzyme 2 (ACE2) has been established, the functional consequences of the initial receptor binding and the stepwise fusion process are not clear. By utilizing a cell–cell fusion system, in complement with a pseudoviral infection model, we found that the spike engagement of ACE2 primed the generation of S2′ fragments in target cells, a key proteolytic event coupled with spike-mediated membrane fusion. Mutagenesis of an S2′ cleavage site at the arginine (R) 815, but not an S2 cleavage site at arginine 685, was sufficient to prevent subsequent syncytia formation and infection in a variety of cell lines and primary cells isolated from human ACE2 knock-in mice. The requirement for S2′ cleavage at the R815 site was also broadly shared by other SARS-CoV-2 spike variants, such as the Alpha, Beta, and Delta variants of concern. Thus, our study highlights an essential role for host receptor engagement and the key residue of spike for proteolytic activation, and uncovers a targetable mechanism for host cell infection by SARS-CoV-2.

## Significance

The SARS-CoV-2 spike protein is responsible for host receptor recognition, membrane fusion, and viral infection. Understanding the cellular and inhibiting the molecular mechanisms of spike-driven viral entry is a research priority in curbing the ongoing pandemic and preventing future coronavirus outbreaks. Here, we highlight that the generation of SARS-CoV-2 S2′ fragments, a proteolytic event occurring within the S2 subunit, is a molecular switch coupled to membrane fusion. Downstream of host receptor recognition, spike-driven syncytia formation requires the presence of an S2′ cleavage site at arginine 815 but not 685. Hence, the proteolytic processing of spike at the S2′ site upon its engagement of host ACE2 may serve as a potential antiviral target against the current SARS-CoV-2 and related coronavirus strains.

The COVID-19 pandemic caused by severe acute respiratory syndrome coronavirus 2 (SARS-CoV-2) infection has exceeded 240 million cases across the globe, but the molecular mechanisms of viral infection and host pathogenesis remain elusive. The SARS-CoV-2 spike (S) glycoprotein is a class I fusion protein decorated on the viral lipid envelope and is a key determinant of viral entry (1). The SARS-CoV-2 spike monomer contains two fragments: The amino terminus S1 subunit contains a receptor binding domain (RBD) (2–5), which recognizes the host receptor angiotensin-converting enzyme 2 (ACE2) for initial docking, while the carboxyl terminus S2 subunit catalyzes the fusion of viral and cell membranes (6, 7), enabling the subsequent release of viral RNA genome and downstream replication within the infected cells (8). Although many studies have captured the stationary phases of spike binding to human ACE2 (9–11), key molecular and cellular processes downstream of receptor recognition have not been explored.

Spike can be proteolytically processed (12). SARS-CoV-2 spike encodes a polybasic cleavage site at its S1/S2 junction, and is posttranslationally processed by the endopeptidase furin (13, 14); cleaved S1 and S2 subunits remain noncovalently attached and fusion competent (15). Furin-cleaved S1 also exposes a C-terminal motif recognized by the host receptor neuropilin-1 (NRP1) (16, 17), which can facilitate SARS-CoV-2 entry. Although spike protein is autoprocessed, additional proteolytic cleavage event within the S2 subunit is proposed to be responsible for the subsequent membrane fusion (18, 19). This cleavage can be mediated at the plasma membrane by the type II transmembrane serine proteases (TMPRSS2) (20–23), or processed by the lysosomal cathepsins during the endocytosis of viral particles (24). Secreted tissue proteases, such as elastase and trypsin, can also facilitate this cleavage event and promote infection (25). As a result, this proteolytic event within the S2 subunit induces the release of a highly conserved hydrophobic region, known as the fusion peptide (18), which subsequently anchors the target host cell membrane (6, 26). A conformational reconfiguration within the S2 subunit then pulls the viral and host membranes into close proximity, allowing lipid membranes to fuse (7, 27–29). The unilateral change of the S2 subunit is of the utmost importance during viral entry, but molecular events regulating the spike processing and activation have not been demonstrated.

Cells infected with SARS-CoV-2 drive the fusion with adjacent ACE2-expressing cells, producing morphologically distinct multinuclear giant cells, also known as syncytia (2, 30, 31). Spike-mediated syncytia have been reported in the postmortem lung samples of severe COVID-19 patients (32, 33). Apart from virus to cell transmission, spike-driven syncytia formation may provide an additional route for cell–cell transmission of SARS-CoV-2. Here, by using a cell–cell fusion system, in complement with a pseudoviral particle infection model, we study the functional and molecular requirements of spike activation. Through analyzing the prefusion and postfusion spike protein products, we show that proteolytic cleavage at the S2′ site is triggered by human cell receptor recognition in a range of immortalized cell lines and humanized primary cells. Generation of the S2′ fragment specifically requires spike recognition of functional host ACE2 and is conserved in the several variants of concern. We highlight that arginine 815, but not arginine residues at the S1/S2 cleavage site, is indispensable for the S2′ cleavage and syncytia formation in wild type (WT), as well as the more infectious Alpha, Beta, and Delta spike variants. Hence, these data highlight that both receptor recognition and proteolytic event at the S2′ site are functionally important for spike-mediated membrane fusion and SARS-CoV-2 infection.

## Results

### Spike-Driven Syncytia Formation Is Coupled to S2′ Fragment Generation in the Presence of ACE2.

**Fig. 1.**
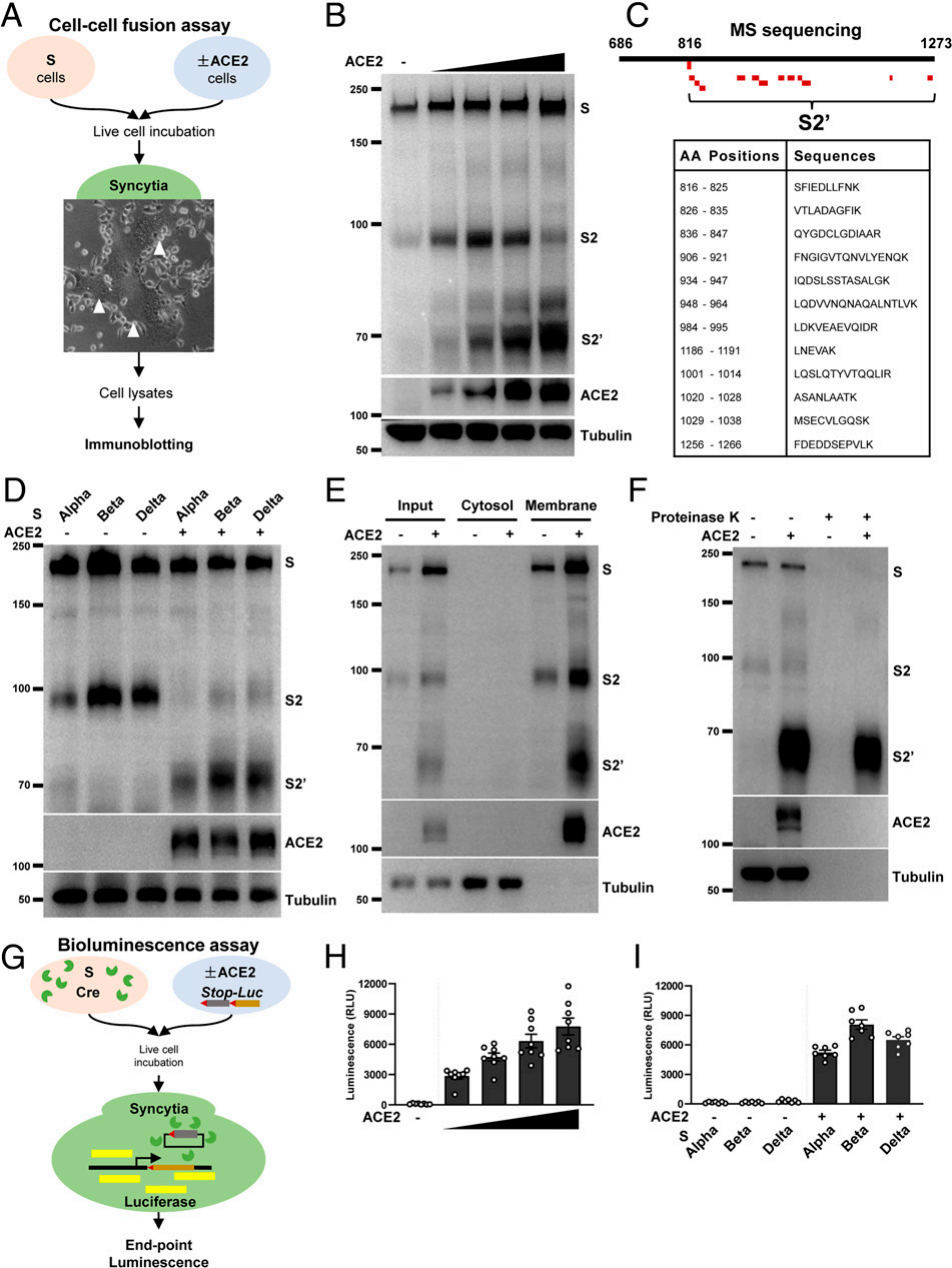
Spike-driven syncytia formation is coupled to S2′ cleavage in the presence of ACE2. (*A*) Schematics of the cell–cell fusion assay using HEK293T cells expressing SARS-CoV-2 spike (S) and ACE2. Live cells were cocultured for 16 h in a humidified incubator at 37 °C before being captured for bright-field or fluorescent images, followed by immunoblotting using standard reducing Tris-glycine SDS/PAGE. (*B*) Immunoblots showing the full-length S, S2, and the cleaved S2′ in relation to ACE2, collected from syncytia cell lysates wherein HEK293T cells expressing S or ACE2 were mixed at various ratios (1:0, 1:0.5, 1:1, 1:1.5, and 1:2), and incubated as described in *A*. Blots are representative of five independent experiments. (*C*) Mapping of SARS-CoV-2 spike peptides detected from mass spectrometry (MS) of the purified S2′ fragment; peptide coverage of the S2 subunit is displayed in red and mapped between amino acid position 816 and 1266. (*D*) Immunoblots showing the S2′ cleavage detected from spike Alpha, Beta, and Delta variants when HEK293T-ACE2 cells were cocultured for 16 h in a cell–cell fusion assay. Blots are representative of three independent experiments. (*E*) Immunoblots showing the membrane association of S, S2, S2′, and ACE2 collected from the syncytia lysates of cocultured HEK293T cells expressing S and ACE2. Blots are representative of three independent experiments. (*F*) Immunoblots showing the proteinase K-sensitive and -resistant species of full-length S, S2, S2′, and ACE2. HEK293T syncytia were lysed in Nonidet P-40 lysis buffer before being treated with or without 10 μg/mL proteinase K for 30 min. Blots are representative of two individual experiments. (*G*) A schematic diagram demonstrating the Cre-loxp system using a *Stop-Luc* reporter system for the bioluminescence detection of syncytia formation; Cre and *Stop-Luc* were cotransfected with S and ACE2, respectively. Luciferase expression and activity from syncytia lysates were quantified as RLU. (*H*) Luciferase activity (RLU) measured from HEK293T cells coexpressing WT S and Cre, cocultured with *Stop-Luc–*expressing cells transfected with increasing amounts of ACE2; cells expressing *Stop-Luc* without ACE2 transfection were used as the negative control. Data shown are representative of eight independent repeats. (*I*) Luciferase activity (RLU) measured from HEK293T cells coexpressing spike Alpha, Beta, and Delta variants and Cre, cocultured with control or ACE2*Stop-Luc*–expressing cells for 16 h. Data shown are representative of six independent repeats. Data are shown as individual points with mean ± SEM.

To investigate the functional and biochemical signatures of spike (S) protein upon engaging its receptor ACE2 in live cells, we first utilized a cell–cell fusion assay to obtain potentially cleaved spike protein products from syncytia (Fig. 1*A*). In this system, HEK293T cells (without endogenous ACE2 expression) transfected with plasmids encoding WT spike readily formed syncytia after coculturing with HEK293T cells expressing human ACE2 (HEK293T-ACE2) and Vero E6-ACE2, as well as colorectal adenocarcinoma Caco-2 and lung adenocarcinoma Calu-3 cells expressing endogenous ACE2 (*SI Appendix*, Fig. S1*A*). We then collected these adherent syncytia and immunoblotted for cleaved spike species using a rabbit polyclonal antibody specifically detecting the S2 ectodomain (686 to 1208) through standard reducing Tris-glycine sodium dodecyl sulfate polyacrylamide gel electrophoresis (SDS/PAGE). This experiment revealed that spike expression in HEK293T cells displayed full-length S and autocleaved S2 fragments at ∼195 and ∼98 kDa, respectively, as reported earlier (14, 15) (Fig. 1*B*). Upon addition of HEK293T-ACE2 cells onto the spike-expressing HEK293T cells, an additional band S2′ at ∼68 kDa was detected, which was absent from spike-expressing HEK293T cells cocultured with control HEK293T cells (Fig. 1*B*). The abundance of S2′ band was dependent on the number of ACE2-expressing cells being added, suggesting that the increasing amount of ACE2 promoted the proteolytic processing of spike (Fig. 1*B*).

To validate the peptide sequence of the S2′, we performed immunoprecipitation of spike protein products from HEK293T syncytia lysates. Purified S2 protein fragments were separated and examined on polyacrylamide gels using silver staining (*SI Appendix*, Fig. S1*B*); the S2′ fragment was then extracted for mass spectrometry analysis (methods described in *SI Appendix*). Within the S2 subunit, 12 unique peptides were detected, listed, and mapped between the spike 816 and 1266 amino acid positions (Fig. 1*C* and *SI Appendix*, Fig. S1*C*), confirming that the S2′ is a further cleaved spike species downstream of S2.

To go further, we tested three SARS-CoV-2 variants of concern, namely, Alpha (Pango lineage B.1.1.7), Beta (B.1.351), and Delta (B.1.671.2), using our cell–cell fusion system. These variants carry multiple forms of mutations spanning different regions of the spike S1 and S2 subunits (*SI Appendix*, Fig. S1*D*), notably, the D614G substitution that promotes the S1 incorporation and infectivity (34–37), and the P681H and P681R substitutions that are adjacent to the S1/S2 furin cleavage site in the Alpha and Delta variants, respectively (38–40). HEK293T cells expressing these spike variants exhibited slightly increased autocleaved S2 in Beta and Delta variants when cocultured with control HEK293T cells without ACE2 (Fig. 1*D*); after adding HEK293T cells expressing ACE2, generation of the cleaved S2′ band at ∼68 kDa was robustly detected in all spike variants, and was increased in Beta and Delta variants when compared with the Alpha variant (Fig. 1*D*). These data indicate that SARS-CoV-2 spike S2 can be autocleaved in the absence of ACE2, whereas S2′ cleavage occurs exclusively in the presence of ACE2-expressing cells.

To examine whether generation of a SARS-CoV-2 S2′ fragment is associated with membrane, we physically homogenized spike-expressing cells and syncytia to separate soluble cytosol and insoluble membranes. S2′ band was only detected from the membrane fractions of the fused HEK293T cells, but not in the cytosolic fraction (Fig. 1*E*), suggesting that the S2′ remained as a membrane-associating fragment. To confirm whether cleaved S2′ functionally formed a homotrimeric coiled-coil six-helix bundle, we measured its resistance to limited proteolysis using proteinase K (19, 41). This experiment revealed that proteinase K digested the full-length S, autocleaved S2, and ACE2 in both nonfused and fused HEK293T cell lysates, whereas the S2′ fragment remained the only species resistant to the proteinase K digestion in the syncytia lysates detected using the anti-S2 antibody (Fig. 1*F*). Hence, the cleaved S2′ fragment functionally formed a rigid spike fusion core, but the autoprocessed S2 and full-length spike expressed in HEK293T cells did not.

To quantitatively validate spike-driven membrane fusion and subsequent blending of the cytosolic contents of donor and acceptor cells, we utilized a bioluminescence assay system wherein a firefly luciferase (*Stop-Luc*) reporter gene is only expressed when Cre excises the *Stop* cassette inside the fused syncytia (Fig. 1*G*). When spike + Cre-expressing HEK293T cells were cocultured with increasing ratio of HEK293T-ACE2 cells carrying *Stop-Luc*, an ACE2-dependent increase in luciferase signal, quantified as the relative luminescence units (RLU) in postfusion HEK293T syncytia, was detected (Fig. 1*H*). Coculturing spike + Cre-expressing cells with control *Stop-Luc* cells without ACE2 expression did not produce any luminescence signal (Fig. 1*H*), suggesting that ACE2 is required for cell–cell fusion. Moreover, the Beta and Delta variant spikes induced significantly increased luminescence when compared with the Alpha variant only when ACE2-expressing cells were added (Fig. 1*I*), confirming that syncytia formation is regulated by the expression of ACE2 in acceptor cells.

### S2′ Site Cleavage Requires Specific Recognition of Functional ACE2.

**Fig. 2.**
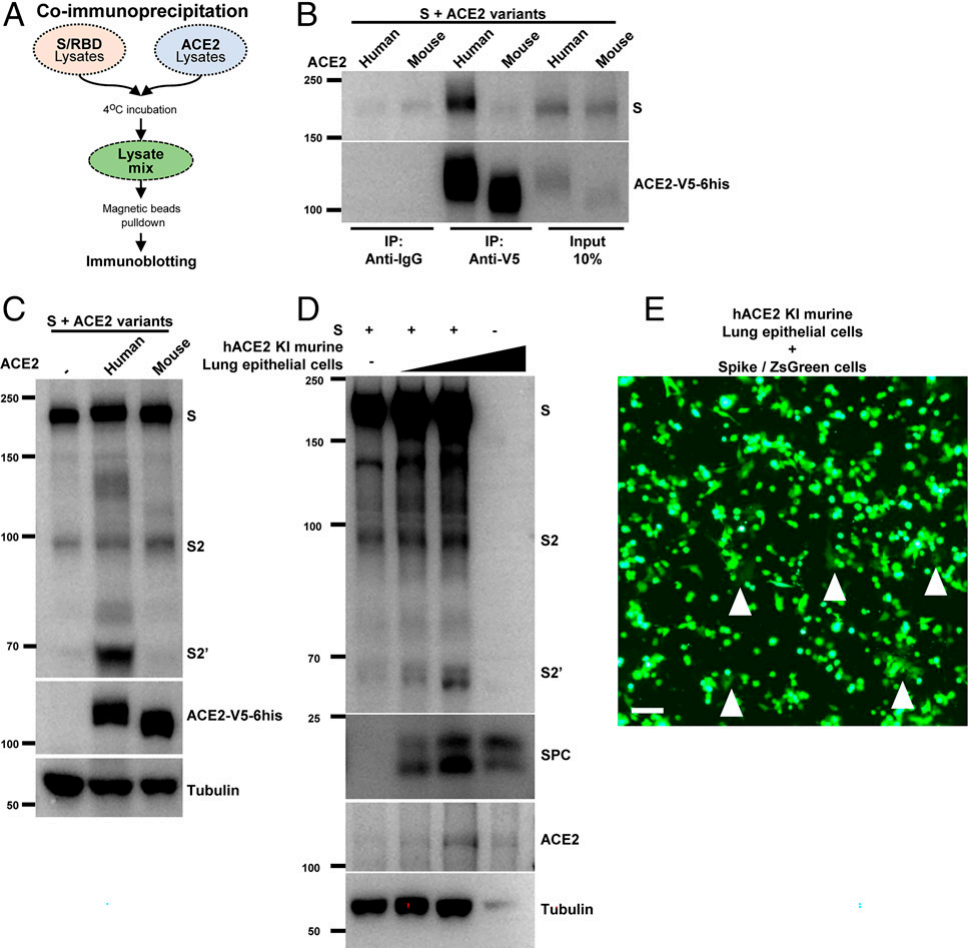
S2′ generation requires specific recognition of functional ACE2. (*A*) Schematics of the coimmunoprecipitation assay using HEK293T cells expressing S or RBD and ACE2. (*B*) Coimmunoprecipitation and (percent of) input controls of full-length spike (S) protein after anti-V5 or anti-IgG pulldown from cell lysates mixed between HEK293T cells expressing human or mouse ACE2-V5-6his and S protein. S was detected using anti-S2 antibody (*Top*), human and mouse ACE2 were detected with anti-his antibody (*Bottom*). Blots are representative of two independent experiments. (*C*) Immunoblots of the cell–cell fusion assay showing the full-length S, S2, S2′, ACE2, and tubulin collected from HEK293T cells expressing S, cocultured with cells transiently expressing human or mouse ACE2-V5-6his for 16 h. ACE2 from different species was detected using anti-his antibody. Blots are representative of four independent experiments. (*D*) Immunoblots of the cell–cell fusion assay showing the full-length S, S2, S2′, ACE2, SPC, and tubulin collected from HEK293T cells expressing S protein, cocultured with primary murine lung epithelial cells carrying human ACE2 and incubated for 16 h. Blots are representative of three independent experiments. (*E*) Fluorescent image of ZsGreen detected in multinuclear fused primary murine lung epithelial cells carrying human ACE2 after coculturing with HEK293T cells coexpressing S and ZsGreen for 16 h. Syncytia are indicated using white arrow heads. (Scale bar, 50 μm.) Image is a representative of two independent experiments.

Mouse ACE2-expressing cells are resistant to WT SARS-CoV-2 spike-mediated infection (3, 42); we therefore examined the specificity of S2′ fragment generation by human and mouse ACE2 variants in the above settings. We designed a coimmunoprecipitation system to validate the mouse or human ACE2 differences, using pulldown approaches by ACE2 attached with C-terminus V5 and 6his tags (Fig. 2*A*). Unlike human ACE2, mouse ACE2 in the HEK293T cell lysates displayed no binding to the RBD domain of the spike (*SI Appendix*, Fig. S2*A*); we also confirmed the interaction of full-length spike with human ACE2, but not mouse ACE2 (Fig. 2*B*). When coculturing human and mouse ACE2-expressing cells with HEK293T cells expressing WT spike at 1:1 ratio, human ACE2-expressing cells readily generated the S2′ band, whereas the counterpart cells expressing mouse ACE2 did not (Fig. 2*C*). As a result, HEK293T cells expressing human ACE2, but not mouse ACE2, developed syncytia when cocultured with spike-expressing cells (*SI Appendix*, Fig. S2*B*).

To simulate spike-driven syncytia formation in primary cells, we generated a human ACE2 knock-in (hACE2 KI) mouse line and performed cell–cell fusion assay ex vivo. We isolated murine lung epithelial cells and performed the cell–cell fusion assay with the spike-expressing HEK293T cells. Although only limited expression of ACE2 was detected from the surface protein C-positive (SPC- positive) lung epithelial cells, addition of these cells to spike-expressing cells readily induced the generation of S2′ bands (Fig. 2*D*). Syncytia formation in these primary lung epithelial cells was also visualized using cells coexpressing spike and a ZsGreen fluorescent reporter (Fig. 2*E*). These data demonstrated that S2′ site cleavage and cell–cell fusion can be induced in primary cells expressing functional ACE2.

### Proteolytic Activation of Virion Spike Requires ACE2-Expressing Cells.

**Fig. 3.**
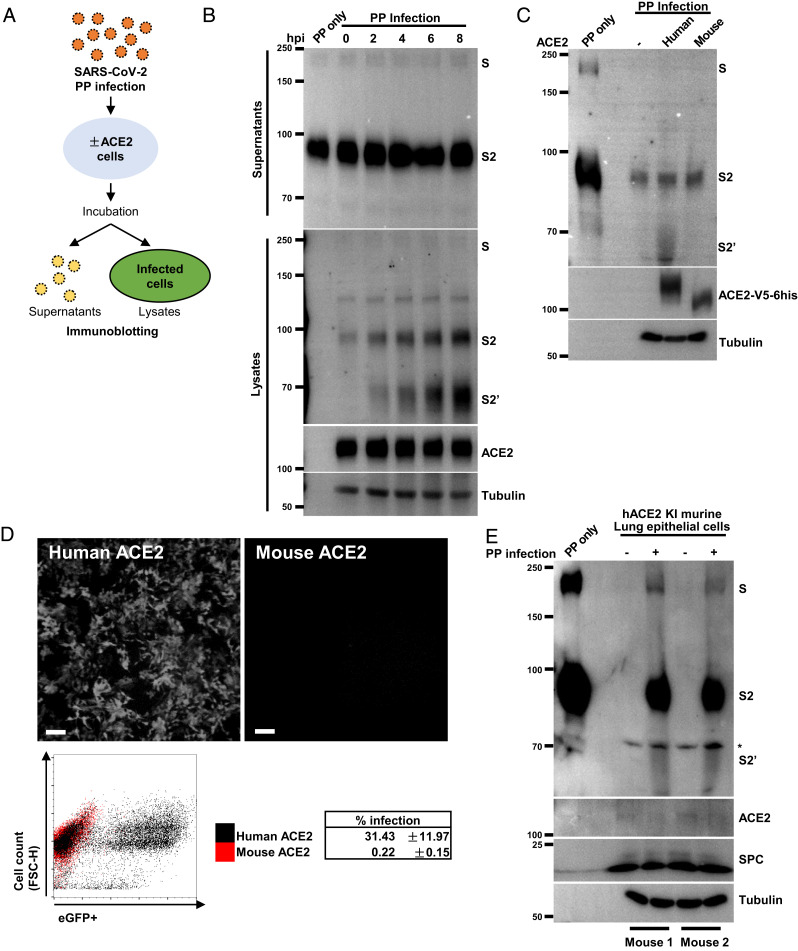
ACE2 primes the proteolytic cleavage of virion spike. (*A*) A schematic diagram illustrating the SARS-CoV-2 PP infection system. HEK293T cells without or with ACE2 expression were incubated with PPs at 37 °C or 4 °C for different time (hours post infection, hpi) as indicated; supernatants or cell lysates were collected for immunoblotting detection of postfusion proteins. (*B*) Immunoblots showing the full-length S, S2, S2′, ACE2, and tubulin detected from supernatants (*Top*) and cell lysates (*Bottom*) from HEK293T-ACE2 cells infected with PP for 0, 2, 4, 6, or 8 h as indicated; negative control was performed by incubating PPs alone at 37 °C for 8 h without cells (lane 1). Blots are representative of three individual repeats. (*C*) Immunoblots showing S, S2, S2′, ACE2 and tubulin, detected from HEK293T cells expressing human or mouse ACE2-V5-6his infected with PPs for 6 h. ACE2 variants were detected using anti-his antibody. Blots are representative of four individual repeats. (*D*) Fluorescent images showing eGFP expression as a result of SARS-CoV-2 PP infection in HEK293T cells expressing human or mouse ACE2-V5-6his (*Top*); representative dot plot from the FACS showing eGFP-positive cells expressing human ACE2 and PP infection rate from three individual experiments was summarized as the mean ± SD (*Bottom*). (*E*) Immunoblots showing the full-length S, S2, S2′, ACE2, SPC, and tubulin detected from cell lysates of hACE2 KI murine lung epithelial cells infected with or without SARS-CoV-2 PPs for 12 h. Asterisk denotes nonspecific bands, blots are representative results obtained from two independent experiments.

To support our observations obtained from cell–cell fusion assays, we utilized an infection model to examine the proteolytic processing of virion spike during viral entry. First, we prepared retroviral pseudotyped particles (PPs) with SARS-CoV-2 WT spike proteins and incubated with HEK293T-ACE2 cells for 8 h, before collecting both supernatants and adherent cells to examine the potentially cleaved spike species during viral entry (Fig. 3*A*). Unlike ectopically expressed in cells, SARS-CoV-2 WT spike protein assembled on virions collected from supernatants after incubation was mainly cleaved into the S2 species detected using the anti-S2 ectodomain antibody, which is in agreement with previous reports (14, 15, 43, 44) (Fig. 3*B*, *Top*). PP in the supernatants remain unchanged 8 h postinfection (hpi) in mock and infected groups (Fig. 3*B*, *Top*). However, an increase of S2 as well as cleaved S2′ band was readily detectable from HEK293T-ACE2 cell lysates during the course of infection, suggesting a time-dependent increase in cleavage during the viral entry (Fig. 3*B*, *Bottom*).

To determine whether S2 or S2′ fragment is associated with PPs infection, we used HEK293T cells transiently expressing human or mouse ACE2-V5-6his, and collected cell lysates 6 hpi for the detection of spike proteolytic products. Generation of the S2′ band was specifically detected from cell lysates expressing human ACE2, but not control cells or cells expressing mouse ACE2 (Fig. 3*C*). As a result, HEK293T cells transiently expressing human ACE2, but not mouse ACE2, were robustly infected by PPs encoding the eGFP (PP-eGFP) reporter 48 hpi (Fig. 3*D*), which is in agreement with our cell–cell fusion assay mentioned above. To test whether S2′ fragment generation occurred as a result of ACE2 binding and downstream proteolysis, we tested PPs infection of ACE2-expressing cells at 4 °C, a suboptimal temperature that prevents downstream viral particle entry. In this case, generation of an S2′ fragment, but not S2 band, was prevented when incubating PPs with ACE2 cells at 4 °C (*SI Appendix*, Fig. S2*C*), suggesting that binding of ACE2 is unable to directly drive S2′ site cleavage, and this proteolysis requires a physiological relevant temperature.

To confirm that proteolytic activation of virion spike occurred in primary cells and tissues, we infected lung epithelial cells from the hACE2 KI mice ex vivo as described above. Spike protein was readily cleaved into the S2′ fragment in primary cells isolated from two mice 12 hpi (Fig. 3*E*). Furthermore, PPs infection of hACE2 KI trachea tissue produced similar proteolytic product (*SI Appendix*, Fig. S2*D*). These data support a converging role for human ACE2 in priming the S2′ fragment cleavage during viral entry.

### Spike Arginine 815 Is Required for S2′ Cleavage and Syncytia Formation.

**Fig. 4.**
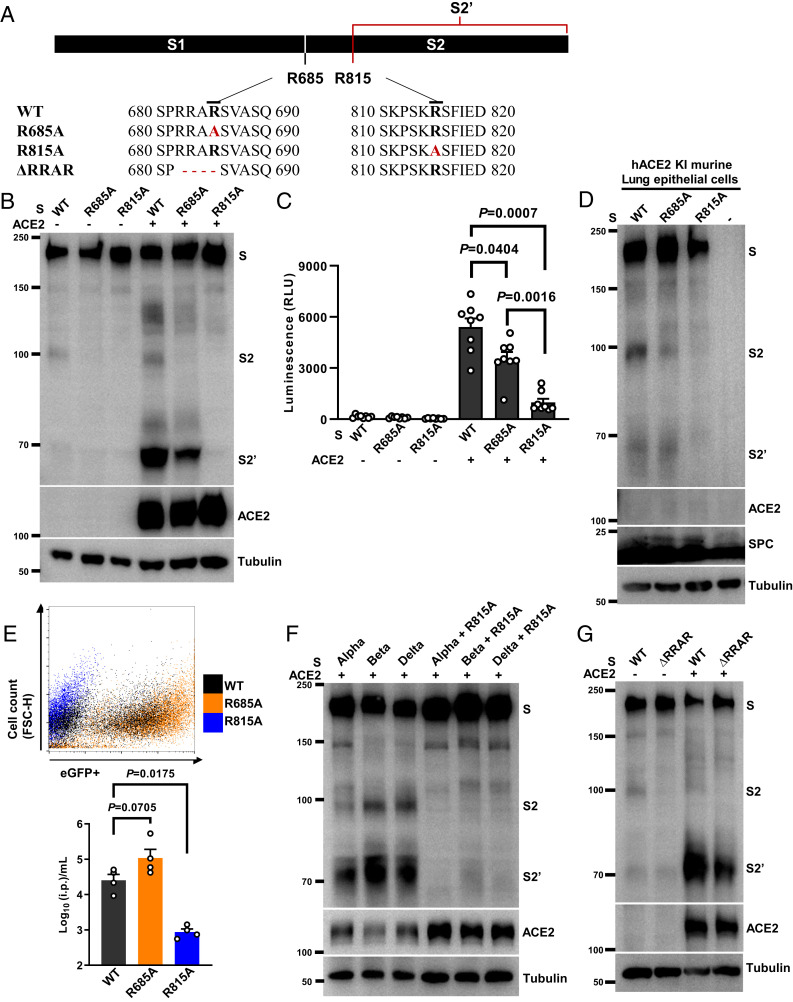
Spike arginine 815 is required for S2′ cleavage and syncytia formation. (*A*) Schematics and amino acid sequences depicting the relative positions of S1/S2 and S2′ cleavage sites along the spike protein; spike R685A and R815A substitutions and ΔRRAR are highlighted in red. (*B*) Immunoblots showing the full-length S, S2, S2′, and ACE2 collected from HEK293T cells expressing S-WT, S-R685A, or S-R815A cocultured with HEK293T cells without or with ACE2 expression for 16 h. Blots are representative of five independent experiments. (*C*) Luciferase activity (RLU) detected from S-WT, S-R685A, or S-R815A induced syncytia lysates. Data are shown as individual points with mean ± SEM from eight independent experiments. (*D*) Immunoblots showing the full-length S, S2, S2′, ACE2, SPC, and tubulin collected from HEK293T cells expressing S-WT, S-R685A, or S-R815A mixed with murine lung epithelial cells isolated from hACE2 KI mice. Blots are representative of two individual experiments. (*E*) Representative dot plot from FACS showing percentage of eGFP-positive HEK293T-ACE2 cells infected by SARS-CoV-2 PPs prepared using S-WT (Gray), S-R685A (Orange), or S-R815A (Blue) for 48 h. Numbers of i.p. per milliliter were calculated from infected HEK293T-ACE2 cells; data summarized from four individual repeats were presented as mean ± SEM. (*F*) Immunoblots showing S, S2, and S2′ generation in HEK293T cells transiently expressing spike Alpha, Beta, and Delta variants containing R815A mutations upon cocultured with HEK293T-ACE2 cells for 16 h. Blots are representative of three individual experiments. (*G*) Immunoblots showing the full-length S, S2, S2′, ACE2, and tubulin from HEK293T cells expressing WT orΔRRAR spike mutants mixed with HEK293T cells without or with ACE2 expression for 16 h. Blots are representative of three individual repeats. *P* values were obtained by one-way ANOVA with Sidak’s post hoc test and are indicated in *C*.

The SARS-CoV-2 spike protein can be cleaved at multiple locations, notably, the cross-strain conserved arginine residues at the S1/S2 junction (685) or S2′ site (815) (14, 15) (*SI Appendix*, Fig. S3*A*). To compare the functional difference between arginine residues at S2 and S2′ sites, we performed single arginine to nonpolar aliphatic alanine substitutions at the spike S1/S2 junction (R685A) or S2′ (R815A) sites (Fig. 4*A*). Neither R685A nor R815A mutants affected the localization of spike protein onto the plasma membrane, as shown by immunostaining of the S1 subunit on the nonpermeabilized HEK293T cells (*SI Appendix*, Fig. S3*B*). This was further confirmed by flow cytometry of the spike-expressing cells, where the detection of S2 on the cell surface was similar between WT (38.8%), R685A (37.8%), and R815A (38.2%) spike-expressing cells (*SI Appendix*, Fig. S3*C*). Both mutant spike proteins were detected in the membrane fractions using biochemical fractionation (*SI Appendix*, Fig. S3*D*), and did not alter the spike interaction with the receptor ACE2 compared with WT spike (*SI Appendix*, Fig. S3*E*).

Of note, however, in a cell–cell fusion assay, spike R685A and R815A mutants expressed in HEK293T cells strongly reduced the autocleaved S2 at ∼98 kDa compared with WT spike-expressing cells (Fig. 4*B*). When ACE2-expressing cells were added, R685A spike mutant-expressing cells produced a noticeably reduced S2′ fragment (Fig. 4*B*), as well as the significantly decreased luminescence signal compared with WT spike (Fig. 4*C*). However, the R815A spike mutant-expressing cells completely prevented the generation of an S2′ band when cocultured with HEK293T-ACE2 cells (Fig. 4*B*), and drastically abolished the luminescence signal, compared with WT and R685A spike mutant-expressing cells (Fig. 4*C*). As a result, we did not detect syncytia formation induced by R815A spike mutant in HEK293T-ACE2, Vero E6-ACE2, or Caco-2 cells (*SI Appendix*, Fig. S4). More importantly, the R815A spike mutant, but not WT or R685A spike mutants, completely prevented S2′ band generation in primary lung epithelial cells isolated from hACE2 KI mice (Fig. 4*D*), which was also evidenced by the lack of syncytia formation, visualized using the spike/ZsGreen reporter cells (*SI Appendix*, Fig. S5*A*). The effect of R815A spike mutant stayed consistent when cell–cell fusion was performed with human lung epithelial Calu-3 cells, where both WT and R685A spike mutants displayed robust S2′ generation, but there was a lack of syncytia formation in the R815A spike mutant (*SI Appendix*, Fig. S6 *B* and *C*).

To extend the role of arginine 815 in mediating viral entry, we prepared retroviral PPs using WT, R685A, and R815A spike mutants. When compared with WT, PPs prepared using R685A spike mutant displayed increased infectious particles (i.p.) per milliliter when infecting HEK293T-ACE2 cells, whereas PPs prepared using R815A spike mutation completely abolished PPs entry into HEK293T-ACE2 cells (Fig. 4*E*). In line with WT PPs, infection of HEK293T cells expressing ACE2 by R685A spike mutant PPs displayed S2′ cleavage 6 hpi (*SI Appendix*, Fig. S6*A*). In addition, substituting R815 to a polar uncharged asparagine (R815N) residue also prevented the generation of an S2′ band in the cell–cell fusion system (*SI Appendix*, Fig. S6*B*), as well as fusion-mediated luminescence signal (*SI Appendix*, Fig. S6*C*), both confirming the role of R815 site on the spike in mediating cell–cell fusion. Consistent with the above findings, PPs prepared with R815N and R815A spike mutants were no longer infectious compared with WT spike in HEK293T-ACE2 cells, indicated with eGFP expression (*SI Appendix*, Fig. S6*D*).

Apart from WT spike, introduction of R815A mutation to Alpha, Beta, and Delta spike variants strongly abolished the S2′ site cleavage (Fig. 4*F*) and syncytia formation (*SI Appendix*, Fig. S6*E*), thus highlighting a conserved cleavage event at the S2′ site. We also generated a spike mutant deficient in S1/S2 cleavage by deleting arginine residues RRAR (ΔRRAR) (Fig. 4*A*). Complete removal of arginine residues at the S1/S2 cleavage site abolished spike autoprocessing by furin, and even in the presence of exogenously trypsin (*SI Appendix*, Fig. S7*A*). Similar to the spike R685A mutant, spike ΔRRAR mutant still produced the S2′ band and syncytia when ACE2-expressing cells were added (Fig. 4*G* and *SI Appendix*, Fig. S7*B*), confirming that S1/S2 cleavage is not essential for the downstream S2′ processing. These data suggest that the arginine 815 residue of the spike protein is important for syncytia formation in cultured cell lines, as well as cells derived from primary tissues.

### ACE2 Primes the S2′ Site Cleavage in a Cell-Free System.

**Fig. 5.**
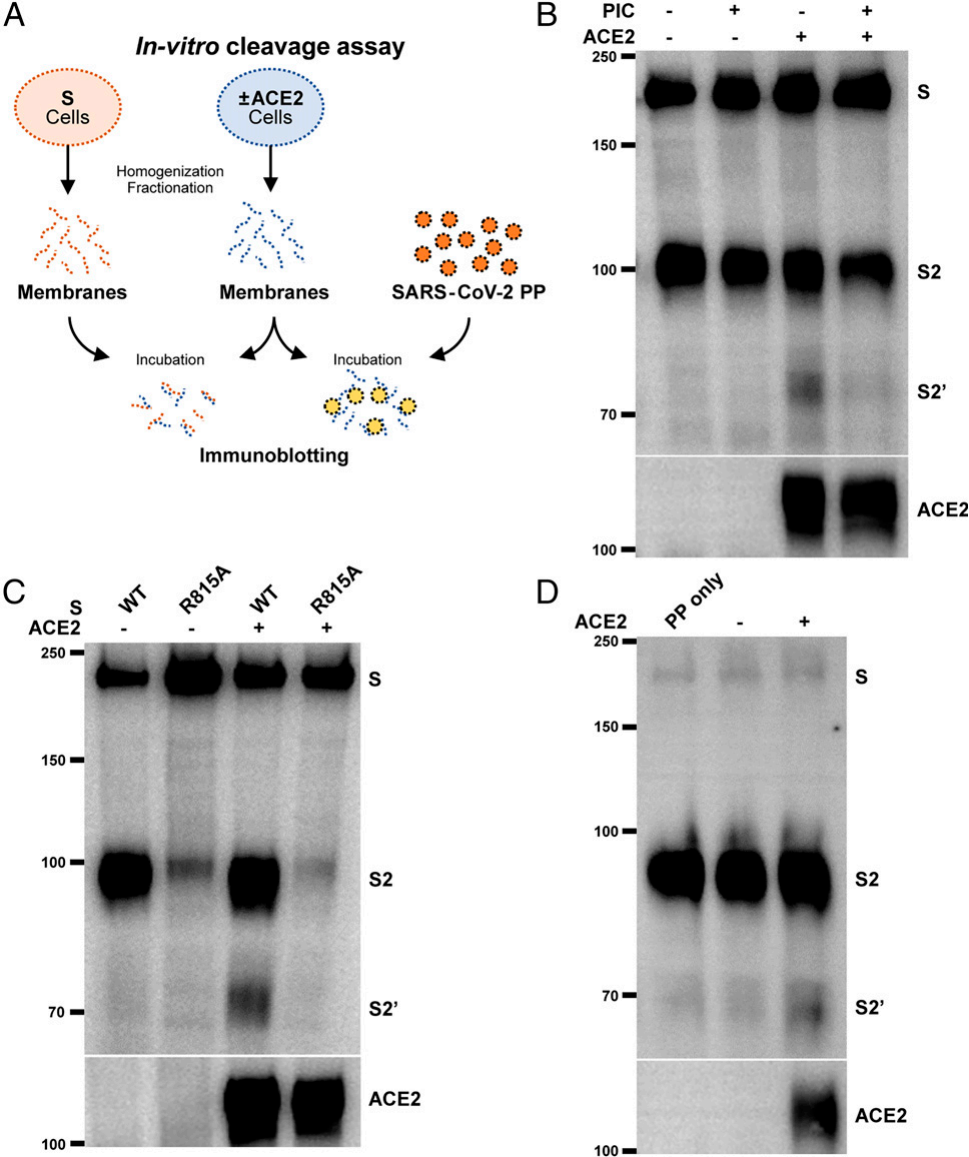
ACE2 primes the S2′ site cleavage in the absence of cellular membrane fusion. (*A*) Schematics illustrating the in vitro spike cleavage assay using the cell membrane fractions. HEK293T cells expressing SARS-CoV-2 spike (S) or ACE2 were homogenized and fractionated for crude cell membranes. Membrane pellets were resuspended and mixed in serum-free DMEM for 16 h in a humidified incubator at 37 °C. Alternatively, cell membranes with or without ACE2 were directly resuspended in media containing PPs. Cell membrane mixes were then harvested for immunoblotting using standard reducing Tris-glycine SDS/PAGE. (*B*) Immunoblots showing the full-length S, S2, S2′, and ACE2 from HEK293T cell membrane mixes. HEK293T cell membranes containing WT spike were incubated with or without ACE2-expressing membranes, in the absence or presence of 1× PIC for 16 h. Blots are representative of three individual experiments. (*C*) Immunoblots showing the specific cleavage of S2′ in HEK293T cell membrane mixes containing WT and R815A spike, incubated with or without ACE2-expressing membranes for 16 h. Blots are representative of three individual experiments. (*D*) Immunoblots showing the full-length S, S2, S2′, and ACE2 from HEK293T cell membrane and PP mixes. PPs prepared with WT spike were mixed with membranes with or without ACE2 expression for 16 h; control PP were incubated in serum-free DMEM without cell membranes. Blots are representative of three individual experiments.

In order to determine whether proteolytic cleavage of spike at the S2′ site occurs prior to membrane fusion, we utilized an in vitro method to examine the cleavage of spike in the isolated cell membranes. HEK293T cells expressing control vector, spike, or ACE2 were physically homogenized, and membrane fractions were obtained as described in Fig. 1*G*; crude membranes were then resuspended in serum-free medium, mixed, and incubated at 37 °C for 16 h to examine the cleavage products of spike in the absence or presence of ACE2 (Fig. 5*A*). When mixing HEK293T cell membranes containing spike and ACE2, a proteolytic cleavage product resembling an S2′ fragment was readily detected from the pellet mix (Fig. 5*B*). Cleavage at the S2′ site was inhibited when similar membrane mix was treated with 1× protease inhibitor mixture (PIC) (Fig. 5*B*), suggesting that a membrane-associated protease is responsible for the generation of an S2′ band. To confirm that spike R815 is specifically cleaved in this system, we included the spike R815A mutant as a noncleavable control. Only ACE2-containing membranes induced a robust cleavage of the WT (Fig. 5*C*), but not the R815A spike mutant, suggesting that an ACE2-induced structural change is required to expose the R815 cleavage site. Moreover, to mimic the proteolytic activation of virion spike protein, PPs were resuspended in HEK293T cell membrane fractions without ACE2 expression. Here, spike protein on the PPs was readily cleaved into the S2′ fragment only in the membranes containing ACE2, but not by membranes without ACE2 expression (Fig. 5*D*). These data suggested that ACE2 primes a specific cleavage at the S2′ site even in the absence of live cells, and protease activity on the membrane is required for spike proteolytic activation.

Since the membrane-associated protease responsible for cell–cell fusion has not been identified in the HEK293T cells, we investigated the potential effect of exogenous trypsin on activating the S2′ cleavage and cell–cell fusion at early time points (12, 18). When a high concentration of trypsin (10 μg/mL) was added to the HEK293T cells expressing the WT spike mixed with control HEK293T cells expressing *Stop-Luc* in the serum-free medium for 3 h or 6 h at 37 °C, no cell–cell fusion or increase in luminescence signal was detected (*SI Appendix*, Fig. S7*C*). This implied that trypsin alone at 10 μg/mL was insufficient for the proteolytic activation of SARS-CoV-2 spike protein and cell–cell fusion. However, addition of HEK293T cells coexpressing ACE2 and *Stop-Luc* readily induced a time-dependent increase in luminescence signal even at 3 and 6 h (*SI Appendix*, Fig. S7*C*); luminescence signal was significantly enhanced by the addition of exogenous 10 μg/mL trypsin at both time points (*SI Appendix*, Fig. S7*C*). In addition, trypsin enhanced the luminescence signal detected from both WT and R685A spike mutants at 6 h, but not in the S2′ R815A spike mutant (*SI Appendix*, Fig. S7*D*). These data suggested that, in the presence of host cell receptor ACE2, activation of spike at the S2′ site fulfilled by a trypsin like protease occurs at an early stage to drive cell–cell fusion.

## Discussion

Pneumocyte syncytia in the postmortem lung tissues has been a hallmark of SARS-CoV-2 infection and is largely attributed to the fusogenicity of spike protein. How pneumocyte syncytia exacerbates lung pathology, or its implications in post-COVID conditions, remains to be understood. Although SARS-CoV-2 mainly infects epithelial linings of the nasal mucosa, trachea, and bronchi (45), these human primary respiratory cells are often difficult to obtain, maintain, and manipulate; expression of ACE2 in these primary tissues is also limited (46), hindering the study on how spike mediates membrane fusion in human cells. By utilizing HEK293T cells, an immortalized cell line deficient in ACE2 expression, we dissected the minimal requirement of spike proteolytic activation in vitro. Our coculture model relies on the expression of S and ACE2 in donor and acceptor cells, respectively, to differentiate the requirement of ACE2; then we utilized spike mutants to examine the critical site for the spike proteolytic activation. Under these settings, our cell–cell fusion model allowed the robust detection of S2′ fragments; a spike proteolytic product subsequently leads to the downstream membrane fusion.

The coculture model in the current study has limitations in addressing a spectrum of host factors involved in the authentic viral entry in the human airway. Tissue-relevant host serine proteases (21), neutrophil elastases and serum thrombin derived from the infiltrating innate immune cells (12, 25), and presence of shedded soluble ACE2 (47, 48) may alter the mode of spike proteolytic activation along the human respiratory tract. Upon infection, tissue epithelia may also participate in the mucosal defense against viral pathogens by expressing antiviral proteins that antagonize the activities of serine proteases (49), or restrict the viral fusion machinery (30, 50). Humoral immune responses may also trigger a receptor mimicry that promotes antibody-dependent enhancement during the later phase of COVID-19 (27). Hence, an in vitro cell line model is unable to reflect the dynamic involvement of host factors during spike-driven membrane fusion, which still requires thorough investigation in a more tissue-relevant context.

Numbers of polybasic residues at the S1/S2 junction vary among coronaviruses, such as SARS-CoV, bat-CoV RaTG13, and Middle East Respiratory Syndrome coronavirus (MERS-CoV) (*SI Appendix*, Fig. S4*A*). Presence of polybasic residues at the spike S1/S2 junction may extend serine protease affinity and tropism, such as in the Alpha and Delta variants, and thus result in an increased viral fitness and spread in human lung-associated epithelial cells (38, 44, 51, 52). Alternatively, furin-cleaved S1/S2 may accelerate the cell–cell fusion process by promoting the NRP1 binding and uncapping the S1 subunit (16, 17). Our R685A (RRAA) spike mutant effectively prevented the posttranslational cleavage by furin, and is similar to furin site mutants ΔPRRA, ΔRRAR, and R682A reported by other groups (14, 31, 44, 53). Here, we demonstrated that the R685A and ΔRRAR spike mutants displayed reduced cell–cell fusion, but still produced the S2′ cleavage fragment detected in several cell lines and primary cells (Fig. 4 *A*–*C*). Our furin mutants also likely abolished the C-end rule recognition by the host receptor NRP1, suggesting a nonessential role of NRP1 during the membrane fusion process. When authentic SARS-CoV-2 is passaged in immortalized cells, such as Vero E6-ACE2, the gene that encodes the spike adaptively removes the furin cleavage site and remains transmittable (54–56). It is currently unclear what is driving the selective pressure of the spike furin site in the human lung epithelia; hence, the role of the S1/S2 cleavage site remains to be explored.

In contrast to the furin cleavage site, the S2′ site is more conserved in other coronaviruses. Both S2* of Mouse Hepatitis Virus spike and S2′ of the MERS spike have functional implications for membrane fusion (13, 57, 58). The S2′ site is next to a hydrophobic region, proposed as the fusion peptide that initiates the membrane tethering process (6). These hydrophobic and negatively charged residues carry unique properties that disrupt phospholipids in vitro (26, 59). Hence, additional models are still required to elucidate the stepwise structural changes leading to fusion peptide exposure, and conformational transitions between the prehairpin and hairpin fusion core. Proteolytic cleavage at the S2′ site seemed to be strictly regulated by receptor binding, and questions remain open on why coronaviruses evolve an internal fusion peptide within the S2 subunit; whether polybasic mutations at the S2′ site would also occur to further extend host tropism.

Nonetheless, although our current model is unable to visualize the host response to viral infection in vivo, in vitro assessment of spike proteolytic activation and syncytia formation may provide a robust method for screening small-molecule inhibitors and host-derived factors targeting this event (33, 60, 61). Hence, therapeutics against host receptor usage, or downstream proteolytic events, may improve disease condition and outcomes of current and future cross-strain coronavirus infections.

## Materials and Methods

### Cells and Animals.

All cell lines were purchased from the National Science & Technology Infrastructure cell bank (http://www.cellbank.org.cn). HEK293T, HEK293T-ACE2, Vero E6-ACE2, and Caco-2 cells were cultured in Gibco Dulbecco's Modified Eagle Medium (DMEM) (GE Healthcare) supplemented with 10% fetal bovine serum (FBS) (Sigma) and 1% penicillin/streptomycin (P/S) (Life Technologies) at 37 °C with 5% CO_2_ in a humidified incubator. Human lung cancer cell line Calu-3 was cultured in Minimum Essential Medium supplemented with 10% FBS and 1% P/S (Procell). All cells were routinely tested for mycoplasma contamination; passages between 4th and 25th were used.

K18 promoter-driven hACE2-Cre-ERT2 knock-in (hACE2 KI) mice with a C57BL/6J background were generated by Cyagen and maintained at the specific pathogen-free facility of the Institute Pasteur Shanghai, Chinese Academy of Sciences (IPS-CAS). Animal use, care, and experimental procedures complied with national guidelines and were approved by the institutional Animal Care and Use Committee at IPS-CAS. For experiments, hACE2 KI mice were killed by asphyxiation with CO_2_, perfused with 10 mL of phosphate-buffered saline (PBS), before lung and trachea tissues were surgically obtained. Primary trachea and lung tissues were incubated with collagenase and dispase (Sigma), disassociated at room temperature for 30 min before being washed with PBS (without (ethylenedinitrilo)tetraacetic acid [EDTA]) and resuspended in DMEM supplemented with 10% FBS and 1% P/S. Primary lung epithelial cells were seeded at 5 × 10^4^ cells per mL before cell–cell fusion or PP infection assays. Trachea tissues were cut open lengthwise, washed twice with PBS, and cultivated in serum-free DMEM and OptiMEM in 1:1 ratio supplemented with 5 µg/mL gentamycin sulfate (Sangon) and 1% P/S for PP infection.

### Reagents and Plasmids.

Bovine pancreatic TPCK-treated trypsin (PI20233) was purchased from ThermoFisher. ACE2 polyclonal antibody (21115-1-AP) was purchased from Proteintech. Rabbit anti-S2 (40590-T62) polyclonal antibody was used for the detection of S2′, S2, and S proteins and mouse anti-S1 (40591-MM42) used for the detection of surface S1 protein were purchased from Sino Biological. Mouse anti-prosurfactant protein C (SPC) antibody (AB3786) was purchased from Merck. Mouse anti-HA (AE008), anti-His (AE003), anti-GFP (AE012), anti-V5 (AE017) tag, and anti-β-tubulin (AC030) monoclonal antibodies were purchased from Abclonal. For coimmunoprecipitations, mouse IgG2b kappa isotype control (400302) antibody from Biolegend was used. Horseradish Peroxidase (HRP) conjugated goat anti-mouse (115-035-003) and anti-rabbit (111-035-003) secondary antibodies were from Jackson Immuno Research.

SARS-CoV-2 spike (Wuhan-Hu-1 strain, GenBank ID QHD43419.1) was homo sapiens codon-optimized and generated de novo into pVAX1 vector by recursive PCR. Alpha, Beta, and Delta variants containing point and deletion mutations were generated using stepwise mutagenesis using spike construct containing the truncated 19 amino acids at the C-terminal. Human and mouse ACE2 attached to a C-terminal V5 and 6his tags were cloned from human and mouse intestinal tissues, respectively, into a pcDNA4.0 vector; due to epitope differences of human and mouse ACE2, anti-his tag antibodies were used to detect the protein expression of ACE2. The pcDNA3.1 construct encoding a spike RBD-6his was kindly provided by Prof. Zhong Huang (IPS, Shanghai, China). Site-directed mutagenesis and deletions were performed using customized primers (synthesized by Sangon and Biosun) and Thermococcus kodakaraensis KOD1 high-fidelity polymerase (Toyobo). Parental methylated DNA was digested using Dpn I, and the resultant PCR products were then transformed into XL10-Gold ultra-competent *Escherichia coli* (Agilent). Plasmids were then extracted using DNA extraction miniprep kits, and DNA concentrations were adjusted using Nanodrop (ThermoFisher).

### Transient Transfection and Cell–Cell Fusion Assays.

For transient transfections, HEK293T cells were seeded in flat bottom 24-well plates at 0.5 × 10^6^ cells per mL overnight. Two hundred fifty nanograms of plasmids encoding SARS-CoV-2 spike mutants or ACE2 variants were packaged in Lipofectamine 2000 (Life Technologies) and transfected for 24 h. For bioluminescence assays, 200 ng of plasmid encoding Cre or *Stop-Luciferase* were cotransfected into S- and ±ACE2-expressing HEK293T cells, respectively. For visualization of primary cell and Calu-3 cell syncytia formation, 100 ng of ZsGreen plasmid was cotransfected with spike variants. HEK293T cells in the 24-well plates were then detached using ice-cold calcium-free PBS in the absence of trypsin and centrifuged at 600 × *g* for 4 min.

For cell–cell fusion assays, cell pellets were resuspended into complete DMEM and mixed with HEK293T-ACE2, Vero E6-ACE2, Caco-2, or Calu-3 cells at 1:1 ratio before adhesion to the 48-well and 96-well plates; cell mixes were incubated for 16 h at 37 °C. For trypsin-induced cleavage of spike protein, HEK293T expressing spike variants were resuspended in serum-free DMEM containing 1% P/S without or with 10 μg/mL TPCK-treated trypsin (PI20233, ThermoFisher) for 1 h at 37 °C; subsequently, 10% FBS containing DMEM was added for 16 h of further incubation. Bright-field and fluorescent images showing syncytia formation were captured at endpoint using a 20× objective and 12-bit monochrome complementary metal oxide semiconductor (CMOS) camera installed on the IX73 inverted microscope (Olympus). Attached cells and syncytia were lysed in a Nonidet P-40 lysis buffer containing 0.5% (vol/vol) Nonidet P-40, 25 mM Tris pH 7.3, 150 mM NaCl, 5% glycerol, and 1× EDTA-free PIC (Roche). For proteinase K digestion, cell lysates were incubated with 10 μg/mL proteinase K (AM2548, Invitrogen) for 30 min at 37 °C; reaction was terminated using 5× reducing Laemmli loading buffer. Firefly luciferase activity was quantified as RLU 1 min after mixing cell lysates containing intracellular luciferase with the Dual-Luciferase reporter assay substrate (E1910, Promega) on a Synergy H1 plate reader (Biotek).

### PPs Preparation and Infection.

The retroviral PPs were generated by cotransfection of HEK293T cells using polyethylenimine with the expression vector encoding the SARS-CoV-2 S protein, bundled with the murine leukemia virus (MLV) core/packaging components and a retroviral transfer vector harboring an enhanced green fluorescent protein (eGFP) reporter. The pVAX1-S vectors encoding WT, R685A, R815N, R815A, or the truncated 19 amino acids at the C terminal were used to produce mutant PPs (62). Supernatants that contained PPs were harvested 48 h posttransfection and filtered through a 0.45-μm membrane before being used for infection assays and Western blotting.

Quantification of PP infection was performed in HEK293T-ACE2 cells seeded at 1 × 10^4^ cells-per-well density in a 48-well plate and infected 24 h later with 10, 50, and 100 μL of PP supernatant to a final volume of 150 μL. After 6 h of incubation, the supernatants were removed, and the cells were incubated in complete DMEM further for 48 h to 72 h at 37 °C. The eGFP expression was determined by flow cytometry (fluorescence-activated cell sorter [FACS]) or immunoblot analysis. PP infections were presented as stacked FACS dot plots; i.p. per milliliter of spike mutant PPs was calculated from numbers of infected HEK293T-ACE2 cells derived from various PP titrations.

For the detection of virion S2’, HEK293T or HEK293T-ACE2 cells were seeded at 1 × 10^4^ cells-per-well density in a 96-well plate overnight, before incubation with 100 μL of PPs for 4 h to 8 h at 37 °C with 5% CO_2_ in a humidified incubator; Calu-3 and primary hACE2 KI murine lung epithelial cells were incubated with PPs for 12 h. PPs incubated at identical condition without cells were used as negative controls. After stimulation, supernatants containing PPs, and the infected adherent cells, were collected separately and boiled in reducing Laemmli buffer SDS/PAGE.

### Immunoblotting and Immunoprecipitation.

Tissue culture plates containing adherent syncytia and cell mixes were directly lysed on ice in 2× reducing Laemmli loading buffer before being boiled at 95 °C for 5 min. Protein samples were separated by standard Tris-glycine SDS/PAGE on 7.5% or 9.5% Tris-glycine polyacrylamide gels. Proteins were then transferred onto 0.45-μm poly(vinylidene difluoride) membranes (Millipore) for wet transfer using Towbin transfer buffer. All membranes were blocked in PBS supplemented with 0.1% Tween20 (PBST) and 2.5% bovine serum albumin (BSA) before overnight incubation in primary antibodies at 4 °C. Blots were labeled with HRP-tagged secondary antibodies (Jackson ImmnuoResearch) and visualized with PicoLight substrate enhanced chemiluminescence solution (Epizyme Scientific). Immunoblot images were captured digitally using a 5200 chemiluminescent imaging system (Tanon) with molecular weight markers indicated.

For coimmunoprecipitation, transiently transfected HEK293T cells were lysed on ice for at least 20 min in a Nonidet P-40 lysis buffer containing 0.5% (vol/vol) Nonidet P-40, 25 mM Tris pH 7.3, 150 mM NaCl, 5% glycerol, and 1× EDTA-free PIC (Roche). Crude lysates were clarified by centrifugation at 16,000 × *g* before mixing with S or ACE2 variants and precleared in the presence of Protein A/G magnetic beads (B23202, Bimake) for 1 h at 4 °C. Input samples were obtained from precleared lysate mixes, and resultant lysates were then incubated with mouse anti-V5 or anti-IgG isotype control antibodies overnight at 4 °C. Pull-down samples on the protein A/G magnetic beads were washed three times in Nonidet P-40 lysis buffer before boiling in 2× Laemmli loading buffer. Proteins were then separated by reducing SDS/PAGE and detected by immunoblotting.

### Membrane Protein Extraction.

Membrane and cytosol protein fractionations were prepared using Membrane and Cytosol Protein Extraction Kit (P0033, Beyotime). Briefly, adherent HEK293T cells and syncytia mixes were harvested and resuspended in Hepes-containing lysis buffer (10 mM Hepes pH 7.4, 100 mM NaCl) supplemented with 2× EDTA-free PIC, or as indicated for the in vitro cleavage assay. After 10 min of incubation on ice, cells were physically ruptured by passing through 25-gauge needle syringes. Total homogenates (input) were collected before nuclei clarification at 500 × *g* for 5 min. Soluble fractions were further centrifuged at 16,000 × *g* for 30 min for the pelleting of crude membranes. Membrane proteins were extracted using detergent containing lysis buffer (Buffer B, 10 mM Hepes, 100 mM NaCl, and 1% TritonX-100) and boiled in 5× Laemmli loading buffer. Total lysate, cytosol, and membrane fractions were loaded for comparison.

### Flow Cytometry Analysis.

HEK293T cells were seeded overnight in 24-well plates at a density of 1.5 × 10^5^ cells per well before transfection with spike mutants for 24 h. Cells were detached and blocked in PBS containing 1% BSA in 5-mL polystyrene round-bottom tubes (Corning) on ice for 30 min. Cells were then stained with 1 µg/mL anti-S2 pAb or rabbit IgG isotype control (AC005, Abclonal) for 1 h. Excess antibodies were washed with PBS, and all cells were subsequently stained with goat anti-rabbit secondary antibodies conjugated with AlexaFluor488 (ThermoFisher) before being fixed in 1% paraformaldehyde (PFA) without membrane permeabilization. Flow cytometry was carried out on FACS Celesta (BD Biosciences), and data analysis was performed with FlowJo 10.4 (LCC).

### Statistics Analysis.

Bar graphs were presented as mean values ± SEM with individual data points. All statistical analyses were carried out with the Prism software v8.0.2 (GraphPad). Data with multiple groups were analyzed using matched one-way ANOVA followed by Sidak’s post hoc comparisons. Statistical significance *P* values were indicated between compared groups and shown on figures.

## Supplementary Material

Supplementary File

## Data Availability

All study data are included in the article and/or *SI Appendix*. Plasmids were deposited at the AddGene with following information: Deposit number: 80522, pVAX1-SARS-CoV-2-S, Plasmid #173049, https://www.addgene.org/173049/; pVAX1-SARS-CoV-2 R685A S, Plasmid #180194, https://www.addgene.org/180194/; pVAX1-SARS-CoV-2 R815A S HA-tag, Plasmid #180195, https://www.addgene.org/180195/; pVAX1-SARS-CoV-2 delRRAR S HA-tag, Plasmid #180196, https://www.addgene.org/180196/.
