# Supplementary material for: SARS-CoV-2 spike engagement of ACE2 primes S2′ site cleavage and fusion initiation
Source: Proc Natl Acad Sci U S A. 2021 Dec 20;119(1):e2111199119. doi: 10.1073/pnas.2111199119 (PMC8740742; doi:10.1073/pnas.2111199119)
Supplement: Supplementary File [file pnas.2111199119.sapp.pdf]

PNAS
www.pnas.org

Supplementary Information for

SARS-CoV-2 spike engagement of ACE2 primes S2' site cleavage and fusion initiation

Shi Yu, Xu Zheng, Bingjie Zhou, Juan Li, Mengdan Chen, Rong Deng, Gary Wong, Dimitri Lavillette, Guangxun Meng

Email: Guangxun Meng: gxmeng@ips.ac.cn

This PDF file includes:

Supplementary Methods
Figures S1 to S7

Supplementary Methods

Silver staining and mass spectrometry

For purification of the S2' protein fragment, $1x10^6$ HEK293T cells transiently expressing S and ACE2 were co-cultured at 1:1 ratio in a T25 flasks. After incubation for 16 hours at 37^oC, syncytia lysates were washed once in cold PBS before lysed in NP-40 lysis buffer supplemented with 1x EDTA-free protease inhibitor cocktail. Post-nuclear syncytia lysates were incubated with 1.5 µg rabbit anti-S2 antibody for 1 hour at 4^oC, before immunoprecipitated with Protein A/G magnetic beads. Pull-down proteins were then washed, eluted and boiled in the 2 x Laemmli loading buffer. Total lysates (Input) and immunoprecipitates (IP) were separated by reducing SDS-PAGE on a 7.5% Tris-glycine gel before rinsed in fixation buffer (50% methanol 5% sodium acetate) for 20 min. The gel was sensitized in 0.02% sodium thiosulfate before reacted with 0.1% silver nitrate for 20 min. The gel was eventually developed in 2% sodium carbonate containing 0.045% formalin before rinsed and captured digitally; the S2' band was cut and subjected to in-gel digestion by 12.5 ng/µL trypsin in 25 mM NH_4HCO_3.

Mass spectrometry (MS) analysis was performed by Shanghai Applied Protein Technology Co. Ltd. Briefly, a quadrupole Orbitrap mass spectrometer (Q Exactive) mass spectrometer coupled to Easy nLC (ThermoFisher) was operated in the positive ion mode. MS survey scans (300-1800 m/z) were obtained using the higher collision-energy dissociation (HCD) method in a data-dependent acquisition mode. Normalized collision energy was 30 eV and the underfill ratio was set as 0.1%. The instrument was run with peptide recognition mode enabled. MS spectra were searched using MASCOT v2.2 (Matrix Science) against SARS-CoV-2 spike using the Uniprot reference P0DTC2; decoy hits were used to control the false discovery rate.

Immunofluorescence and confocal microscopy

HEK293T cells were seeded overnight onto sterilized poly-D-lysine (100 ug/mL) (Sigma) treated 10 mm coverslips in 24-well plates at a density of 1.5×10^5 cells per well. After transfection with spike mutants, cells were washed with cold PBS once before fixed with 4% (w/v) paraformaldehyde (PFA) for 20 min. Fixed cells were blocked in 1% (w/v) goat serum in PBS without membrane permeabilization. Anti-SARS-CoV-2 S1 monoclonal antibody in 0.1% (w/v) goat serum in PBS was incubated for 1 hour at room temperature. Coverslips were then washed 3 times with PBS before incubation with goat anti-mouse AlexaFluor555 secondary antibodies (ThermoFisher) for 1 hour at room temperature; secondary-antibody-only controls were performed. Coverslips were washed 3 times before being mounted in neutral mounting medium (Xinyu). Fluorescent images covering various areas on the coverslips were captured at 14-bit depth in monochrome using a 60x oil immersion objective on the Olympus FV-1200 inverted

confocal microscope and subsequently processed using imageJ software (NIH) with scale bars labeled.

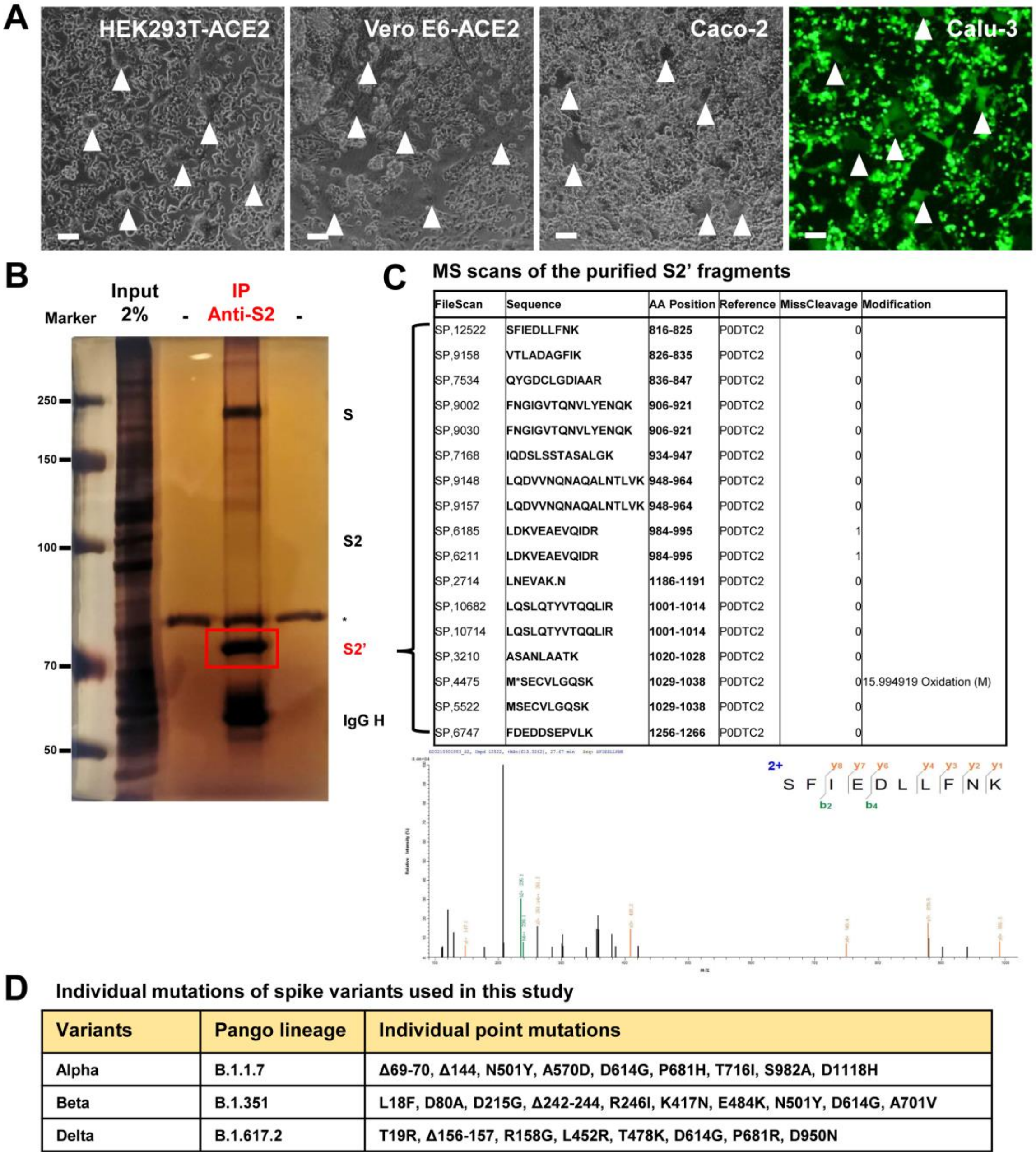

FileScan	Sequence	AA Position	Reference	MissCleavage	Modification
SP,12522	SFIEDLLFNK	816-825	P0DTC2	0	
SP,9158	VTLADAGFIK	826-835	P0DTC2	0	
SP,7534	QYGDCLGDIAAR	836-847	P0DTC2	0	
SP,9002	FNGIGVTQNVLYENQK	906-921	P0DTC2	0	
SP,9030	FNGIGVTQNVLYENQK	906-921	P0DTC2	0	
SP,7168	IQDSLSSTASALGK	934-947	P0DTC2	0	
SP,9148	LQDVVNQNAQALNTLVK	948-964	P0DTC2	0	
SP,9157	LQDVVNQNAQALNTLVK	948-964	P0DTC2	0	
SP,6185	LDKVEAEVQIDR	984-995	P0DTC2	1	
SP,6211	LDKVEAEVQIDR	984-995	P0DTC2	1	
SP,2714	LNEVAK.N	1186-1191	P0DTC2	0	
SP,10682	LQSLQTYVTQQLIR	1001-1014	P0DTC2	0	
SP,10714	LQSLQTYVTQQLIR	1001-1014	P0DTC2	0	
SP,3210	ASANLAATK	1020-1028	P0DTC2	0	
SP,4475	M*SECVLGQSK	1029-1038	P0DTC2	0	15.994919 Oxidation (M)
SP,5522	MSECVLGQSK	1029-1038	P0DTC2	0	
SP,6747	FDEDDSEPVLK	1256-1266	P0DTC2	0	

Variants	Pango lineage	Individual point mutations
Alpha	B.1.1.7	Δ69-70, Δ144, N501Y, A570D, D614G, P681H, T716I, S982A, D1118H
Beta	B.1.351	L18F, D80A, D215G, Δ242-244, R246I, K417N, E484K, N501Y, D614G, A701V
Delta	B.1.617.2	T19R, Δ156-157, R158G, L452R, T478K, D614G, P681R, D950N

Figure S1, Spike-mediated syncytia formation requires species specific recognition of ACE2. (**A**) Brightfield images showing syncytia formation 16 hours after HEK293T cells expressing S-WT mixed with HEK293T-ACE2, Vero E6-ACE2 and Caco-2 cells; fluorescent image of HEK293T-S-WT / ZsGreen cells fused with Calu-3 cells. Scale bars are indicative of 50 µm, images are representative of three independent experiments. (**B**) Silver staining image of full-length S, S2 and S2' immunoprecipitated from HEK293T syncytia lysates. Extracted S2' fragment is indicated in red and subjected to mass spectrometry analysis; Image is a representative of two independent experiments. (**C**) Summary list and a representative MS spectrum of S2' peptides detected from MS scans, data is a representative of two independent MS experiments. (**D**) List of

individual point mutations of the SARS-CoV-2 spike Alpha, Beta and Delta variants used in this study.

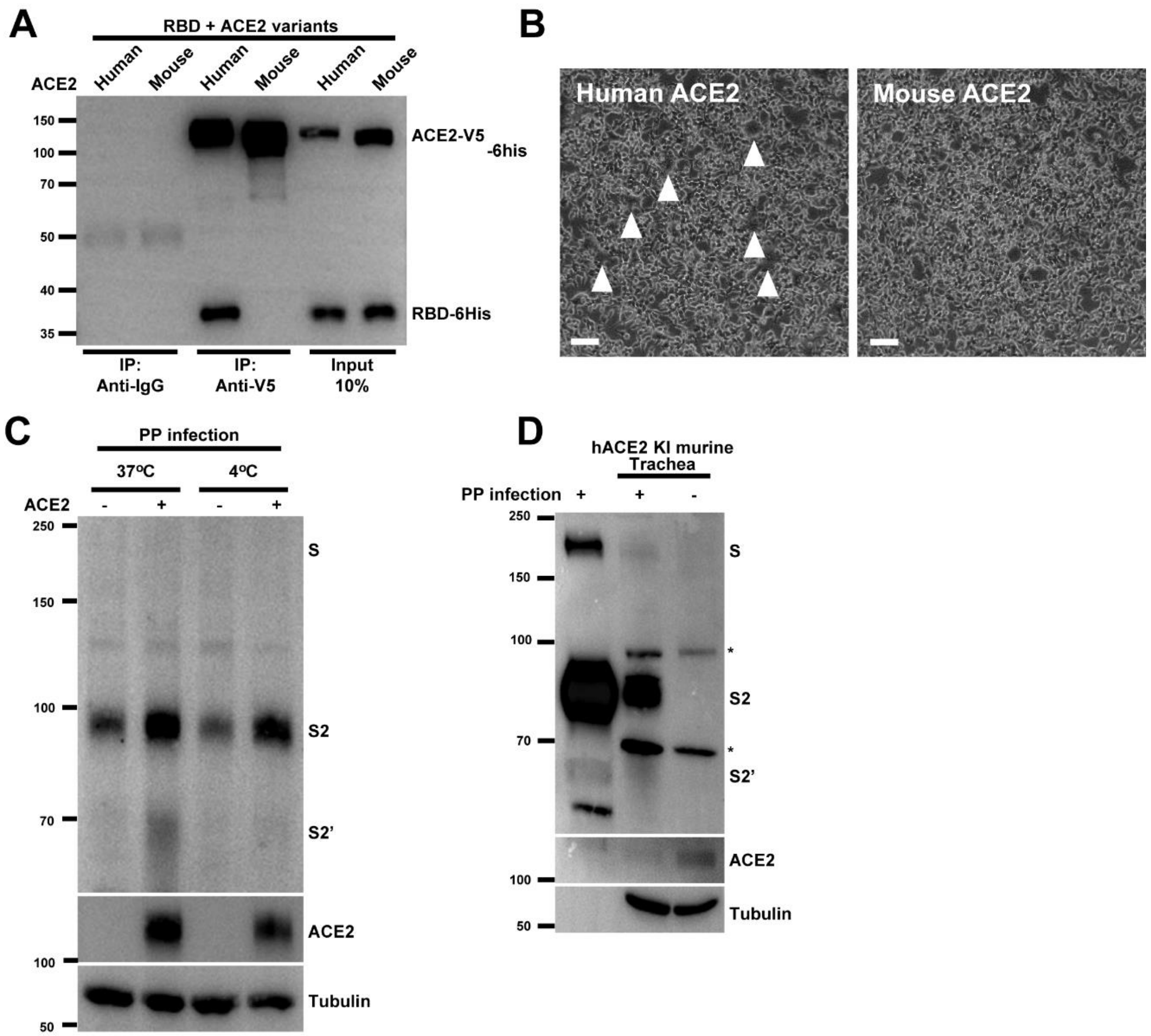

Figure S2, Spike recognition of functional ACE2 is required for S2' cleavage, syncytia formation and PP infection. (**A**) Co-immunoprecipitation and input controls of RBD-6his and ACE2 after anti-V5 or anti-IgG pulldown from cell lysates mixed between HEK293T cells expressing human or mouse ACE2-V5-6his and HEK293T cells expressing RBD-6his. RBD and ACE2 variants were detected using anti-his tag antibody. Blots are representative of two independent experiments. (**B**) Bright field images showing syncytia formation 16 hours after HEK293T cells expressing S-WT, co-cultured with HEK293T cells expressing human or mouse ACE2-V5-6his variants; multinuclear syncytia are denoted with white arrow heads, scale bars are indicative of 50 μm. (**C**) Immunoblots showing full-length S, S2, S2', ACE2 and tubulin from HEK293T cells with or without ACE2 expression, these cells were infected with SARS-CoV-2 PPs for 6 hours post-infection (hpi) at 37°C or 4°C as indicated. Blots are representative of two individual repeats. (**D**) Immunoblots showing full-length S, S2, S2', ACE2 and tubulin collected from hACE2 KI murine trachea tissue lysates infected with SARS-CoV-2 PPs for 12 hours. *denotes non-specific bands, blots are representative of three individual repeats.

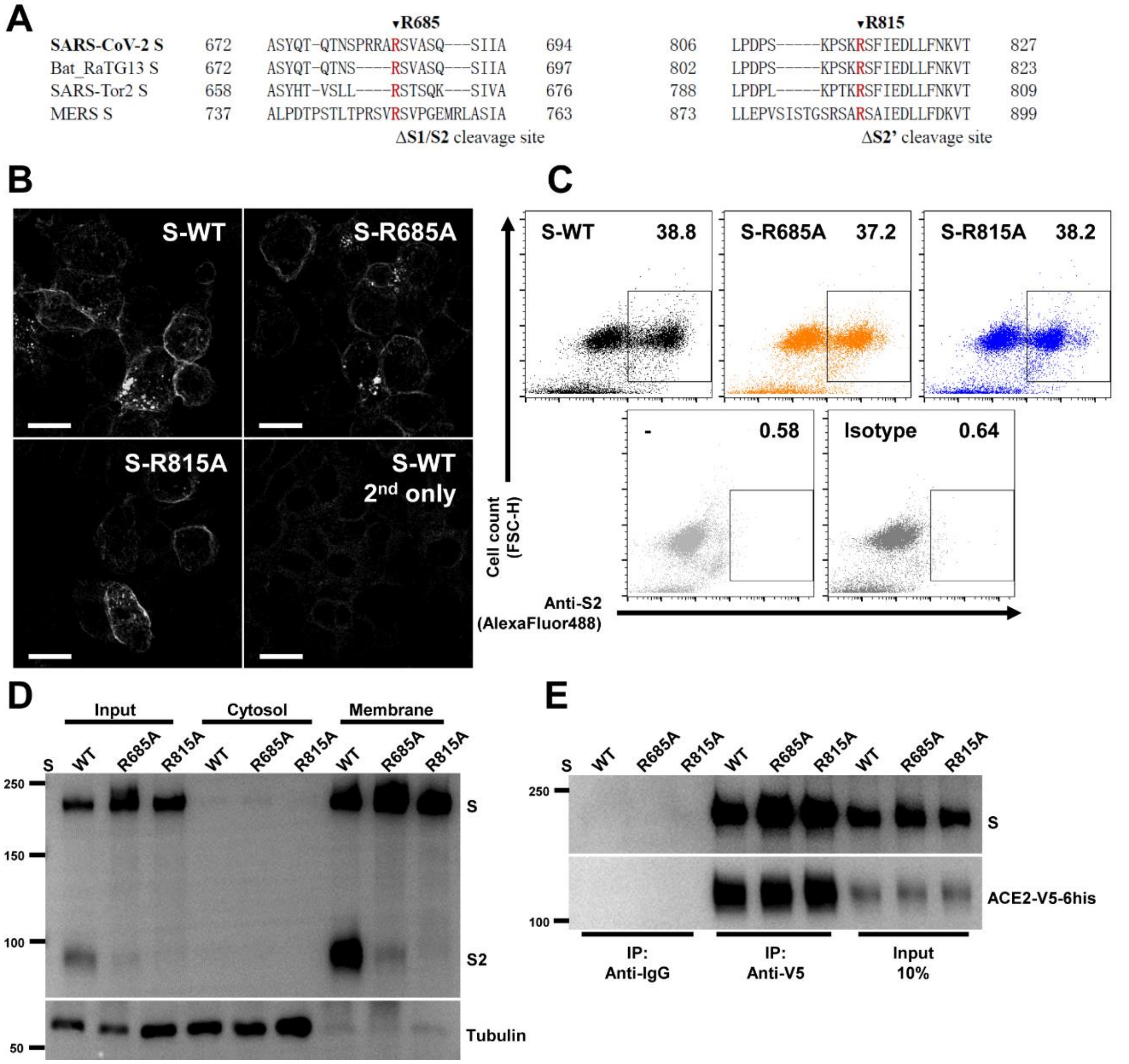

Figure S3, Spike S2 and S2' mutants are localized on the plasma membrane after expression. (**A**) Amino acid sequence alignment of coronaviruses S proteins at the SARS-CoV-2 R685 and R815 loci. Conserved S1/S2 cleavage site arginine residue is highlighted in bold and red; putative cleavage sites are indicated by dark arrow heads. (**B**) Representatives of confocal projections from cell surface immunostaining of S1 in HEK293T cells expressing S-WT, S-R685A and S-R815A mutants. Scale bars are indicative of 20 μm and images are representative of two independent experiments. (**C**) Representative FACS plots showing gating strategy and percentage of positive fluorescent signals (AlexaFluor488) detected from membrane staining of S2 on HEK293T cells (FSC-H) transiently expressing S-WT, S-R685A and S-R815A. **-** denotes control HEK293T cells without S expression. Data are representative of two individual experiments. (**D**) Immunoblots showing the membrane association of S and S2 in HEK293T cells expressing S-WT, S-R685A and S-R815A. Blots are representative of two independent experiments. (**E**) Co-immunoprecipitation and input controls of full-length S after anti-V5 or anti-

IgG pulldown from cell lysates mixed between HEK293T cells expressing human or mouse ACE2-V5-6his and HEK293T cells expressing S-WT, S-R685A or S-R815A mutants. S was detected using anti-S2 (upper panel) antibody, or ACE2 protein detected with anti-ACE2 (lower panel) antibody, blots are representative of two independent experiments.

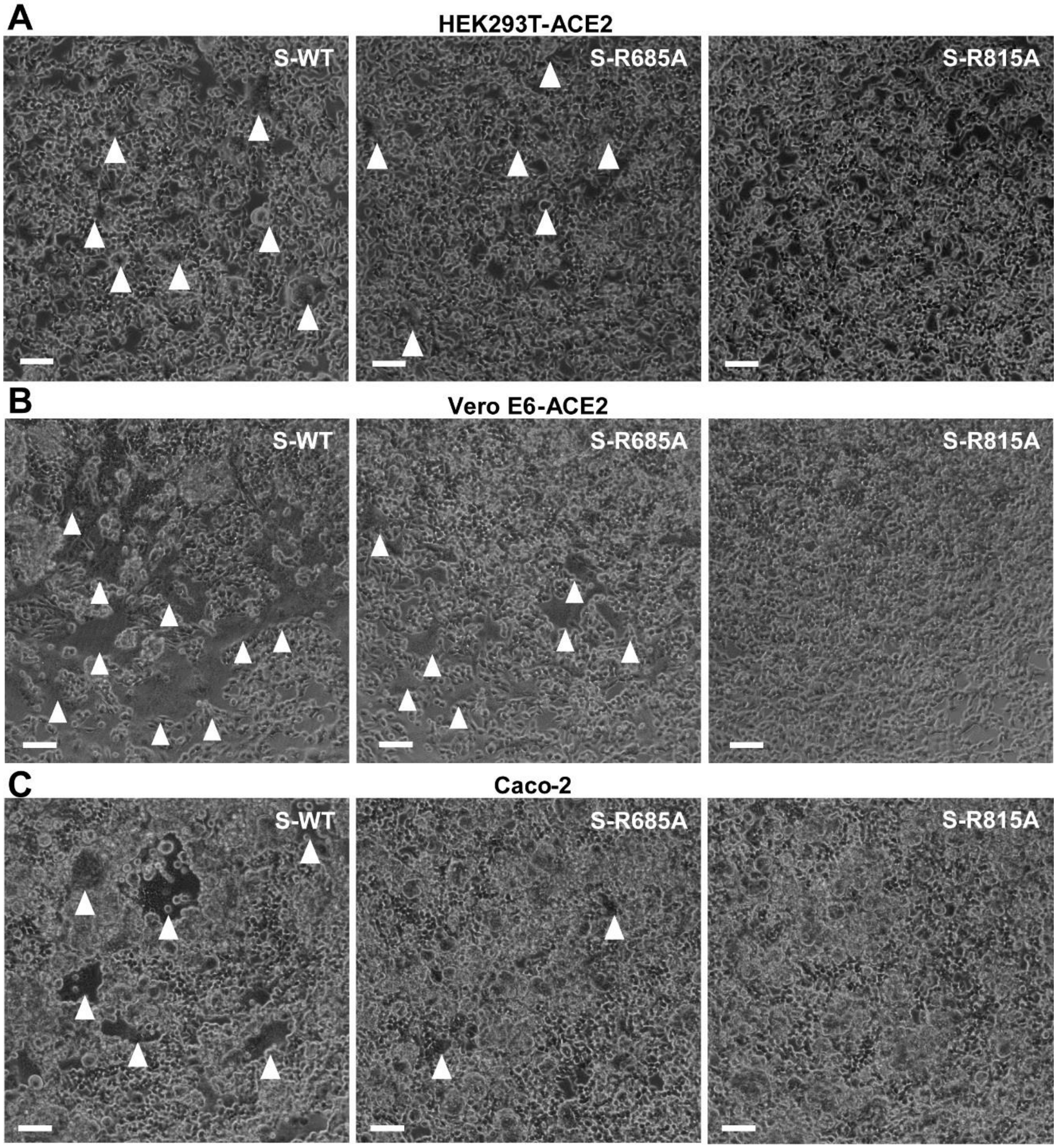

Figure S4, Spike arginine 815 is essential for syncytia formation in various cell lines expressing human ACE2. Representative bright field images showing syncytia formation 16 hours after HEK293T cells transiently transfected with S-WT, S-R685A and S-R815A mixed with HEK293T-ACE2 (**A**), Vero E6-ACE2 (**B**), and Caco-2 (**C**) cells. Multinuclear syncytia are denoted with white arrow heads, scale bars are indicative of 50 µm, images are representative of at least two independent experiments.

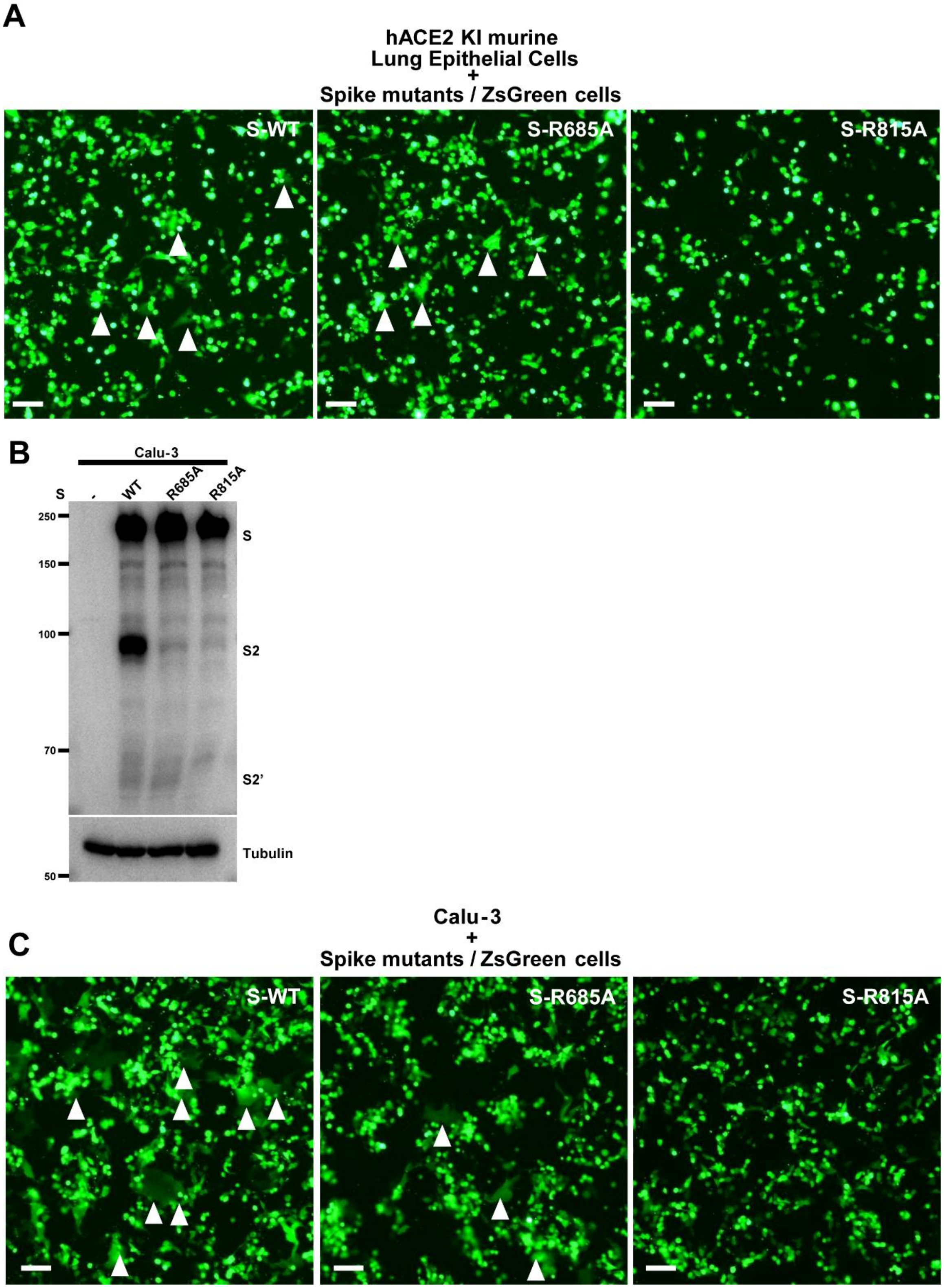

Figure S5, Spike arginine 815 is essential for syncytia formation in primary hACE2 KI murine cells and human Calu-3 cells. (**A**) Fluorescent images of ZsGreen showing syncytia formation in hACE2 KI murine lung epithelial cells co-cultured with HEK293T cells expressing S-

WT, S-R685A or S-R815A co-transfected with ZsGreen plasmid. (**B**) Immunoblots showing the full-length S, S2, S2' from HEK293T cells expressing S-WT, S-R685A or S-R815A co-cultured with human lung Calu-3 cells. Blots are representative of three individual repeats. (**C**) Fluorescent images of ZsGreen for the detection of Calu-3 syncytia formation with HEK293T cells expressing S-WT, S-R685A or S-R815A co-transfected with ZsGreen plasmid. Multinuclear syncytia are denoted with white arrow heads, scale bars are representative of 50 μm, images are representative of at least two independent experiments.

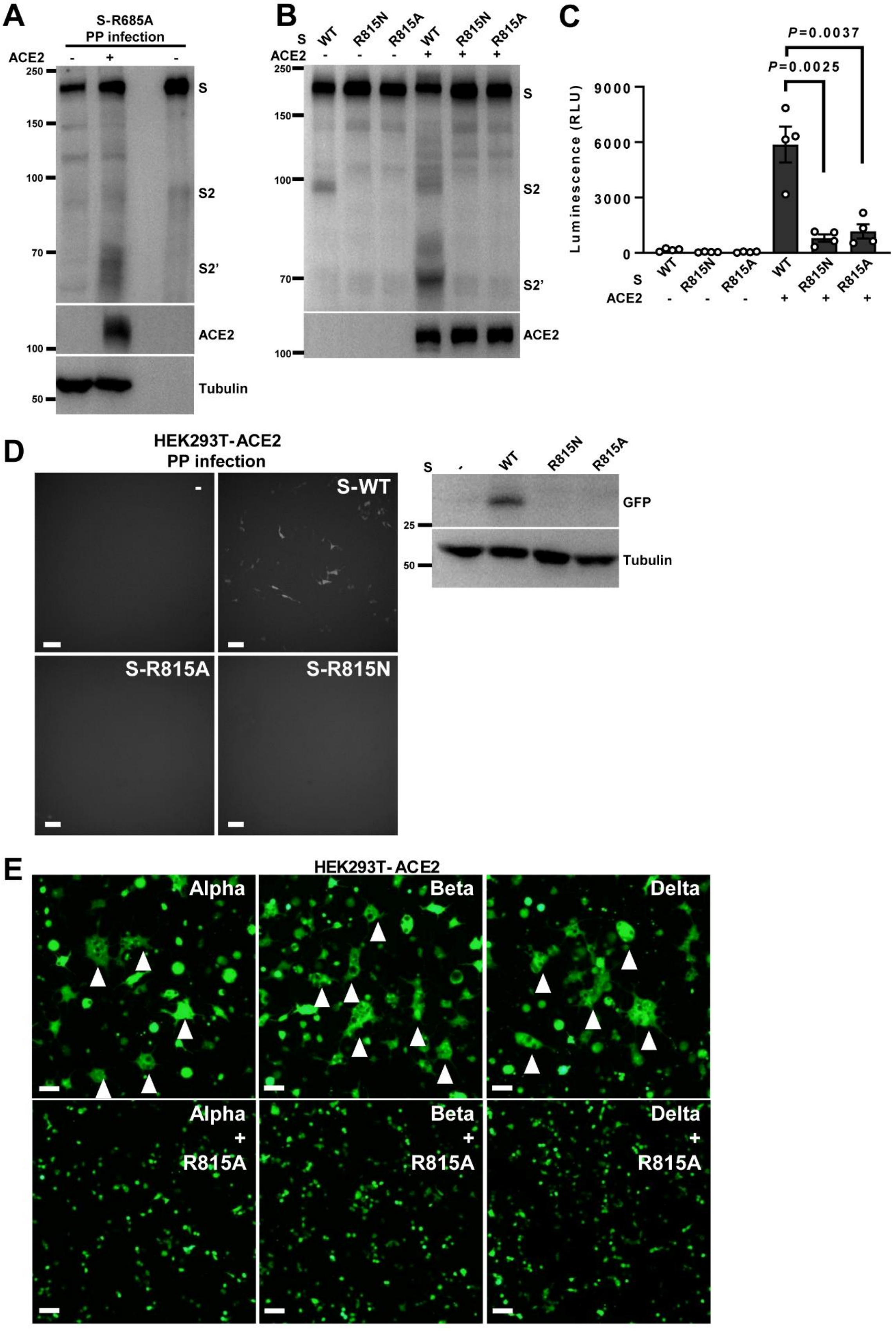
A
S-R685A
PP infection
ACE2
S
S2
S2'
ACE2
Tubulin
B
S
WT
R815N
R815A
WT
R815N
R815A
ACE2
S
S2
S2'
ACE2
C
P=0.0037
P=0.0025
Luminescence (RLU)
9000
6000
3000
0
S
WT
R815N
R815A
WT
R815N
R815A
ACE2
D
HEK293T-ACE2
PP infection
S-WT
S-R815A
S-R815N
S
WT
R815N
R815A
GFP
Tubulin
E
HEK293T-ACE2
Alpha
Beta
Delta
Alpha + R815A
Beta + R815A
Delta + R815A

Figure S6, Spike arginine 815 is essential for syncytia formation and viral infection. (A) Immunoblots showing the full-length S, S2, S2', ACE2 and tubulin detected from HEK293T cells infected for 6 hours by PP prepared using the spike R685A mutant. Blots are representative of three individual repeats. (**B**) Immunoblots showing the full-length S, S2, S2' and ACE2 from HEK293T cells transiently expressing S-WT, S-R815N or S-R815A co-cultured with HEK293T-ACE2 cells for 16 hours. Blots are representative of four independent experiments. (**C**) Luciferase activity (RLU) measured from HEK293T cells co-expressing S-WT, S-R815N or S-R815A and Cre, co-cultured with *Stop-Luc*-expressing cells transfected with ACE2; cells expressing *Stop-Luc* without ACE2 transfection were used as the negative control. Data shown are mean ± SEM from four independent experiments. *P* values were obtained by one-way ANOVA with Sidak's *post hoc* test and are indicated on figures. (**D**) Fluorescent images showing eGFP-positive HEK293T-ACE2 cells infected with PPs prepared using S-WT, S-R815N, or S-R815A mutants, scale bars are representative of 50 μm (left panel). PPs infected HEK293T-ACE2 cell lysates were collected for immunoblots for eGFP expression (right panel). Images and blots are representative of three independent experiments. (**E**) Fluorescent images showing syncytia formation from HEK293T cells co-expressing a ZsGreen reporter and spike Alpha, Beta and Delta variants with or without the R815A mutation, co-cultured with ACE2-expressing cells for 16 hours. Multinuclear syncytia are denoted with white arrow heads, scale bars are indicative of 50 μm, images are representative of at least three independent experiments.

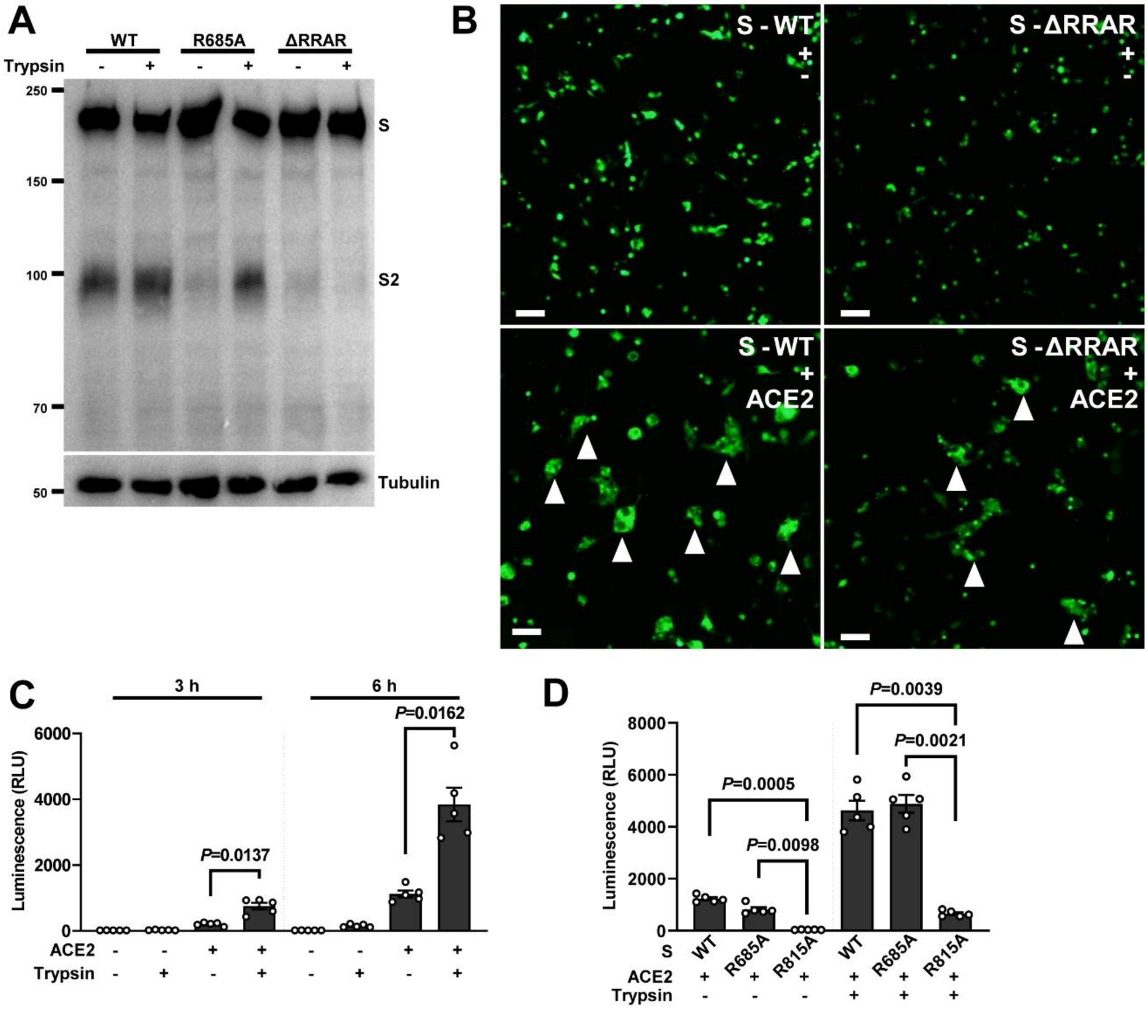

Figure S7, Trypsin enhances spike-driven cell-cell fusion in the presence of ACE2. (**A**) Immunoblots showing trypsin-cleaved the full-length S and S2 collected from HEK293T cells transiently expressing S-WT, S-R685A and S-ΔRRAR mutants, treated without or with 5 μg/mL trypsin for 1 hour. Blots are representative of two individual experiments. (**B**) Fluorescent images showing syncytia formation from HEK293T cells co-expressing a ZsGreen reporter and spike with or without ΔRRAR mutation, incubated with ACE2-expressing cells for 16 hours. Multinuclear syncytia are denoted with white arrow heads, scale bars are indicative of 50 μm, images are representative of at least three independent experiments. (**C**) Luciferase activity (RLU) detected from cell-cell fusion lysates, treated without or with 10 μg/mL trypsin for 3 and 6 hours using the system described in Figure 1G. Data are shown as individual points with mean ± SEM from five independent experiments. (**D**) Luciferase activity (RLU) detected from S-WT, S-R685A or S-R815A spike variants induced cell-cell fusion lysates in the absence or presence of 10 μg/mL trypsin for 6 hours, employing the system described in Figure 1G. Data are shown as individual points with mean ± SEM from five independent experiments. *P* values were obtained by one-way ANOVA with Sidak's *post hoc* test and are indicated on figures.
